# Osteopathic intervention in chronic non-specific low back pain: a systematic review

**DOI:** 10.1186/1471-2474-14-129

**Published:** 2013-04-09

**Authors:** Paul J Orrock, Stephen P Myers

**Affiliations:** 1 School of Health and Human Sciences, Southern Cross University, PO Box 157, Lismore, NSW 2481, Australia; 2 NatMed Research, Southern Cross University, PO Box 157, Lismore, NSW 2481, Australia

**Keywords:** Systematic review, Osteopathy, Osteopathic manipulative treatment, Low back pain, Chronic low back pain, Non-specific low back pain, Manual therapy, Clinical trial methodology

## Abstract

**Background:**

Chronic Non Specific Low Back Pain (CNSLBP) is a common, complex and disabling condition that has been present for longer than three months and is not caused by a serious pathology. Osteopaths are health practitioners who commonly diagnose and treat CNSLBP patients using a complex set of interventions that includes manual therapy. The study aimed to complete a Systematic Review of clinical research into osteopathic intervention in CNSLBP using a rigorous assessment of study quality.

**Methods:**

The literature was searched to August 2011 using the following databases: AMED, CINAHL Plus, Cochrane Central Register of Clinical Trials, MEDLINE Plus, EMBASE, MANTIS, OSTMED, PED*ro*, ScienceDirect. Multiple search terms were used in various combinations: osteopathy/osteopathic, osteopathic manipulative technique, OMT, Spinal Manipulative Therapy, SMT, clinical trial, back pain, chronic back pain. The inclusion criteria were papers that: reported clinical trials; had adult participants; tested the effectiveness and/or efficacy of osteopathic manual therapy intervention applied by osteopaths, and had a study condition of CNSLBP. The quality of the papers was assessed using the Cochrane Back Review Risk of Bias criteria. A meta-analysis would proceed if the studies had adequate clinical and methodological homogeneity.

**Results:**

Initial searches revealed 809 papers, 772 of which were excluded on the basis of abstract alone. The remaining 37 trial papers were subjected to a more detailed analysis of the full text, which resulted in 35 being excluded. The two remaining trials had a lack of methodological and clinical homogeneity, precluding a meta-analysis. The trials used different comparators with regards to the primary outcomes, the number of treatments, the duration of treatment and the duration of follow-up.

**Conclusion:**

There are only two studies assessing the effect of the manual therapy intervention applied by osteopathic clinicians in adults with CNSLBP. One trial concluded that the osteopathic intervention was similar in effect to a sham intervention, and the other suggests similarity of effect between osteopathic intervention, exercise and physiotherapy. Further clinical trials into this subject are required that have consistent and rigorous methods. These trials need to include an appropriate control and utilise an intervention that reflects actual practice.

## Background

### Rationale

Chronic Low Back Pain (CLBP) is pain that is located between the costal margin and buttocks and has persisted for longer than 3 months. Patients suffer physical disabilities and psychological distress concurrently with the pain [1]. The condition has a high incidence and prevalence. International back pain researcher Gordon Waddell [2] described CLBP as a 21st century epidemic. In 2007, 13.8% of Australian population (2,846,400) stated they had a back pain/problem, and/or a disc disorder [3]. These disorders are categorised as musculoskeletal conditions, and in 2004–05, musculoskeletal conditions were more prevalent than any other of the National Health Priority Areas (NHPAs), with 31% of Australians suffering from one or more of these conditions [3]. Arthritis and musculoskeletal conditions were also responsible for the main disabling condition in more than one in three Australians with a disability [4], and were a major area of health expenditure in 2001–02, with around $4.6 billion spent on the conditions.

Non-specific low back pain is described in a recent review of national guidelines [5] as a diagnosis of exclusion, where pain caused by a suspected or confirmed serious pathology (‘red flag’ conditions such as tumour, infection or fracture) or presenting as a radicular syndrome have been ruled out [5]. The review states that some guidelines, e.g. the Australian and New Zealand guidelines, do not distinguish between non-specific low back pain and radicular syndrome.

Osteopathic Medicine is a medical system of diagnosis and therapy based on a set of overarching principles that give osteopathic medicine a holistic basis for its practice [6]. It is practiced worldwide, predominantly in developed western nations, and the practice varies from full medical scope in the US to allied/adjunctive health in the UK, Australia and New Zealand amongst others. A major foundation of osteopathic medicine worldwide is an evaluation of the somatic tissues for signs of dysfunction which is treated with a broad range of manual therapies and adjunctive care.

Osteopaths manage a range of patients depending on the jurisdiction and scope of practice. Because of utilising the holistic diagnostic model and a broad range of manual techniques, Osteopathic Manipulative Treatment (OMT) cannot be confined to a single intervention. Osteopathic medicine is one of the registered professions legally allowed to use Spinal Manipulative Therapy (SMT), defined as manual loading of the spine using short or long leverage methods [2], and SMT as a single modality has been heavily researched [7,8]. John Licciardone, principal author of the only systematic review of OMT in chronic low back pain and a senior clinical academic, warns that OMT is not chiropractic or simple SMT, but a complex intervention based on a multi-factorial diagnostic work up [9].

The results of a sample of 2238 patients presenting to 255 Australian osteopathic practices [10] demonstrate that chronic low back pain is a common presenting problem to these practices, and that the interventions are multi-dimensional. The most common primary presenting symptom was pain located in the lumbar spine (27.3%), and 51.2% of the primary presenting complaints were classified as chronic. The osteopathic intervention on this subset of patients was predominantly soft tissue techniques (78% received this modality), joint articulation (65%), muscle energy (58%), high velocity manipulation (synonymous with SMT) (55%) and exercise advice (42%) [10].

The results of a pilot study surveying 342 osteopathic practices in the United Kingdom that collected data on 1630 patients [11] demonstrated that pain located in the lower back was the most common presenting symptom (36%), and that 37.7% of patients presenting had chronic complaints. The most common osteopathic interventions for these patients were soft tissue techniques (78% received this modality), joint articulation (72.7%), high velocity manipulation (37.7%) and education (35.8%).

There is a need to evaluate the effectiveness of this service to these patients using rigorous research that can be applied to practice. A comparative review of the clinical trial literature of SMT or massage or osteopathy in the treatment of low back pain reveals an evidence base for SMT and massage, both modalities in use by osteopaths, but a lack of research into whole osteopathic practice as demonstrated in the survey data mentioned. A Cochrane review of SMT in low back pain concluded that despite over 800 publications addressing this issue, evidence for the effect on low back pain is equivocal [8]. The Cochrane review of 13 clinical trials of massage found that there is evidence that it may be beneficial for subacute and chronic low back pain in conjunction with exercise [12]. A systematic review and meta-analysis of osteopathic clinical trials up to 2003 [9] concluded that patients had significant improvements from osteopathic intervention, but that many of the results are from trials with small numbers and the intervention is often a single modality or technique.

The question that arises is what is the clinical trial evidence for the osteopathic intervention in CNSLBP, and does the research translate into clinical practice by testing the intervention as it is applied in the everyday practice? Osteopathic intervention for this study is defined specifically as manual intervention and lifestyle advice applied by an osteopath which would be considered by the osteopathic community to be consistent with osteopathic practice. An updated systematic review is warranted to include more recent studies, to apply a rigorous risk of bias assessment, and also to examine the evidence of authentic multidimensional osteopathic intervention, and not simply extrapolating from single modality evidence.

### Objective

This current Systematic Review of clinical research into osteopathic intervention in chronic non-specific low back pain aims to focus on the quality of the evidence and its applicability to practice. Factors underpinning this objective are: to focus on a study condition that commonly presents to this professional group, to use a rigorous mainstream assessment of quality, and finally to review studies that reflect what is known of authentic osteopathic practice.

## Methods

### Eligibility

The inclusion criteria for the initial search were papers that: reported clinical trials, tested the effectiveness and/or efficacy of an osteopathic manual therapy intervention and had a study condition of low back pain. Additional inclusion criteria for the final analysis were: adult subjects; authentic (multidimensional) OMT as the intervention; osteopath as the practitioner; and a study condition of chronic non-specific low back pain.

### Search process

The literature was searched up to August 2011 using the following databases: AMED, CINAHL Plus, Cochrane Central Register of Clinical Trials, MEDLINE Plus, EMBASE, MANTIS, OSTMED, PED*ro*, ScienceDirect. Multiple search terms were used: osteopathy/osteopathic, osteopathic manipulative technique, OMT, Spinal Manipulative Therapy, SMT, clinical trial, back pain, chronic back pain, in various combinations. The reference lists of all articles were searched for other studies, and authors of incomplete or unpublished articles were contacted up till December 2011 requesting details of their trials.

### Study selection

The process of selection followed a broad search to identify trials in SMT as well as OMT, because of the possible overlap in the interventions. Abstracts were read to exclude duplication and trials with no relationship with osteopathy. Studies that appeared to test the research question were then subjected to an analysis of the full text. Included trials were then subjected to a formal Systematic Review.

### Data collection and risk of bias analysis

The two authors independently used data extraction tables and risk of bias analysis based on the Systematic Review Guidelines of the Cochrane Back Review Group [13]. The extraction tables described each study’s design, participants, randomisation, level of blinding, drop out rate, inclusion and exclusion criteria, treatment, control group, outcome measures and results. The risk of bias criteria are outlined in Table 1 – one point was given for each affirmative answer. Before commencing the formal review, the two authors tested their rating consistency by independently reviewing an unrelated clinical trial paper and discussed any minor differences in their interpretation of the guidelines.

**Table 1:** **Assessment for sources of risk of bias **[13]

1	Randomisation adequate?	Yes/No/Unsure
2	Concealed treatment allocation?	Yes/No/Unsure
3	Patient blinded?	Yes/No/Unsure
4	Care provider blinded?	Yes/No/Unsure
5	Outcome assessor blinded?	Yes/No/Unsure
6	Drop out rate described?	Yes/No/Unsure
7	Participants analysed within group?	Yes/No/Unsure
8	Free of selective outcome reporting?	Yes/No/Unsure
9	Groups similar at baseline?	Yes/No/Unsure
10	Co-interventions avoided/similar?	Yes/No/Unsure
11	Compliance acceptable?	Yes/No/Unsure
12	Timing of outcome similar?	Yes/No/Unsure

### Synthesis of results

The study results would be pooled and a meta-analysis performed following the guidelines in the Cochrane Handbook for Systematic Reviews of Interventions [14]. For this study, the factors established for inclusion in a meta-analysis were that the studies had homogeneity in subjects, study condition, intervention and outcome measures.

The a priori criteria for determining the superiority (and inferiority) of one treatment compared to another would need to demonstrated by statistical significance (α<0.05) in a high quality randomised appropriately controlled study. Non-inferiority and equivalence would have needed to have been demonstrated in a study specifically designed to determine these effects with appropriate statistical analytical methods [15]. In the situation that multiple studies existed they would be subject to a meta-analysis. If the studies were too heterogeneous to warrant a meta-analysis; then the evidence for superiority,non-inferiority or equivalence would be assessed for consistency across the studies. Where the studies were equivocal, weighting would be given to those studies with higher methodological validity and statistical power.

## Results

### Study selection

**Figure 1:**
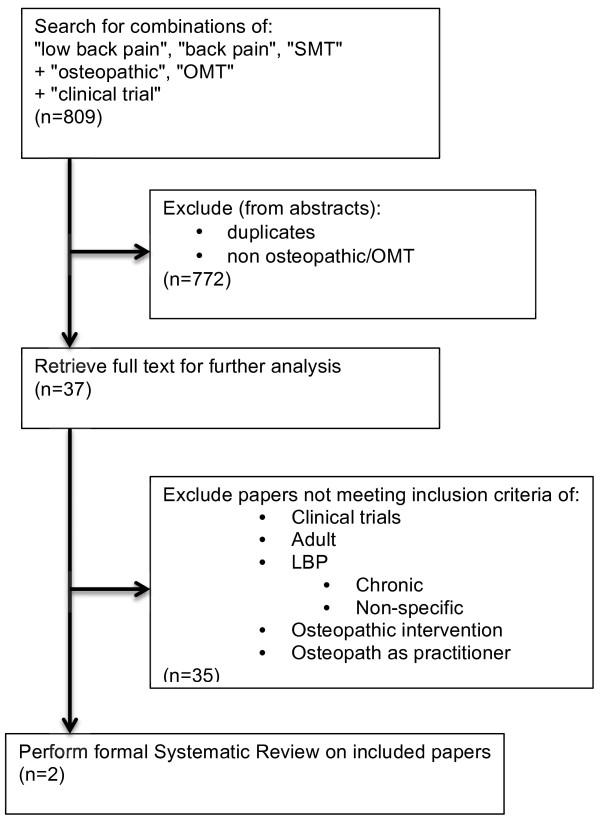
Flowchart of paper selection.

The initial search terms and article elimination processes are outlined in Figure 1. The authors of one unpublished trial were contacted but the results were not released for this review due to the publication peer review being currently underway. After the application of the specific inclusion criteria, 2 articles remained.

### Study characteristics

The two articles that remained are summarized in Table 2. There was one study added to the most recent Systematic Review in 2003 [9]. Randomisation was consistently applied, but there was a range of outcome measures and intervention characteristics. The Licciardone et al. study [16] used the SF-36 as the primary outcome measure, whereas the Chown et al. study [17] used the Oswestry Disability Index. The package of osteopathic manual interventions that were applied with discretion by the treating osteopaths were similar, but were applied at different intensities and frequencies.

**Table 2:** Characteristics and risk of bias assessment of included trials

**Author**	**Licciardone, 2003 **[21]	**Chown et al., 2008 **[26]
Participants	N=91, 21-69yo	N=239, 18-65yo
Randomised	Yes	Yes
Blinding	Patients	Patients and Assessors
Inclusion	NSLBP for 3 months	NSLBP for 3 months
Exclusion	Red flags, neurological signs, surgery, workers comp, pregnancy, recent manipulation	Red flags, radiculopathy, surgery, anti-coagulants
Intervention detail	Senior osteopathic students	One osteopath
	Choice of soft tissue, MET, Art, HVT, SCS, cranio-sacral, myofascial technique	Choice of soft tissue, MET, Art, HVT, functional, exercise, education, psychosocial, nutritional advice
	Seven sessions over 5 months	Five sessions over 3 months
	Follow up at 1, 3 and 6 months	Follow up 6 weeks and 12 months
Control	Sham or no treatment	Manipulative PT or group exercise
Outcome measures	SF-36, VAS, RM, ODI, satisfaction questionnaire	ODI, EuroQoL, Shuttle walk test, satisfaction questionnaire
Main results	SF-36:	(For osteopathy only)
	1 month OMT >control (p=0.03)	ODI - 5.0 (95% CI 1.6 – 8.4; SD 10.5; p<0.01):
	3 months Sham > OMT/control (p=0.01)	EQ-5D 0.11 (CI 0.02 to 0.19; SD 0.24; p<0.05):
	6 months Sham > OMT/control (p=0.03)	Group comparison not done
	VAS pain:	
	1 month OMT/Sham >control (p=0.01/0.003)	
	3 months OMT/Sham >control (p=0.001/0.001)	
	6 months OMT/Sham >control (p=0.02/0.02)	
	RM no differences	
	OMT less co-treatments (p=0.03)	
Risk of Bias score /12 Detail of point loss	7	9
	Randomisation process not fully described	Patients not blinded
	Care provider not blinded	Care provider not blinded
	Drop out rate not fully described	Compliance not acceptable
	Co-interventions not avoided	
	Compliance not acceptable	
Quality Issues	Confounders in sham techniques, co-treatments	Sample size reduced
		Statistical analysis incomplete

### Risk of bias within studies

The included studies were analyzed for risk of bias and the results are summarized in Table 2. The scores ranged from 7/12 to 9/12, demonstrating they had a low risk of bias [13], with the criteria most commonly lacking dealing with blinding of those involved, and a lack of patient compliance.

### Results of individual studies

The two trials included which investigated osteopathic manual interventions by osteopathic clinicians in chronic non-specific lower back pain in adults differed in their conclusions: one concluded that the osteopathic intervention was similar in effect to a sham intervention [16], and the other suggests similarity of the effect of osteopathic intervention with exercise and physiotherapy [17].

The trials used different comparators with regards to the primary outcomes, the number of treatments, the duration of treatment and the duration of follow-up. Licciardone et al. [16] compared osteopathic intervention to sham intervention (range of motion, light touch and simulated techniques) and a non-treatment; the primary outcome was the Medical Outcomes Study Short Form-36 Health Survey (SF-36); provided seven treatments over five months; and assessed the difference between baseline and six months. Chown et al. [17] compared osteopathic intervention to group exercise and physiotherapy; the primary outcome was the Oswestry Disability Index (pain intensity and effects on daily living); provided five treatment sessions to be undertaken within a three month duration; and assessed the difference between baseline and 6 weeks; and six weeks and follow-up at 72–79 weeks.

The Licciardone et al. trial [16] had a systematic exclusion procedure ensuring the patients had non-specific lower back pain. Whilst it was reported that applicants were excluded if they had ever been a patient at the trial clinic site, it was not reported whether the subjects had any previous experience of OMT which could affect their blinding with regards to the sham control. The randomisation process was performed using sequentially sealed envelopes in order to establish a balance of OMT and non-OMT groups, but this was not fully described – it was unclear whether this was this a system of alternates for each subject allocation, or that the envelopes were shuffled between allocations, or some other method. As the authors discussed, the osteopathic assessor and treating practitioners were pre-doctoral students and this relative inexperience may have affected the result. The drop out/attrition rate was relatively high at 27.5%, and the specific reasons for this attrition were only given for two dropouts from the intervention group. The usual care group used more co-treatments - these were not described and could have included medication, physical and exercise therapy, massage and other interventions that are similar to the OMT intervention, and as the authors mentioned, may have attenuated its effects.

The Chown et al. trial [17] attracted a large recruitment base but had a significant attrition, especially in the exercise group. The first two interventions appeared to be delivered by single therapists, which reduces the generalizability of the results to the broader professional groups. The subjects were not blinded to the interventions, and it was unclear whether they knew the difference between physiotherapy and osteopathy, nor whether they had experienced these interventions before.

### Synthesis of results

The heterogeneity between these two studies meant that the planned meta-analysis was not performed because of both clinical and methodological diversity [14]. While there is significant differences between these two trials, one trial concluded that the osteopathic intervention was similar in effect to a sham intervention, and the other suggests similarity of effect between osteopathic intervention, exercise and physiotherapy. This is in contrast to the Systematic Review of 2003 that found that OMT significantly reduced low back pain compared to controls [9]. The authors of both included studies recommend that larger scale clinical trials be undertaken with care given to recruitment and retention of participants and also to the experience of the treating clinicians.

## Discussion

### Summary of evidence

These findings clarify the current state of research on the effect of the osteopathic intervention in the treatment of chronic non-specific lower back pain in adults. This condition in the adult population has been identified as one of the most prevalent presentations to osteopathic clinicians; and as such, needs to be seen as a key research priority for the osteopathic profession. The results of this review suggest that there is a paucity of quality clinical trials that assess the effectiveness of osteopathic medicine intervention in this condition in the adult population, with only two trials included. These suggest that OMT appeared similar to sham intervention [16], and exercise and physiotherapy [17].

This review differs in its conclusions from the previous Systematic Review of 2003 [9], which concluded there was a positive effect from OMT in patients with low back pain. There were a number of trials included in the 2003 review that were not included in the current one for a number or reasons. The Hoehler [18], Gibson [19] and Andersson [20] trials did not fit the criteria of testing only chronic low back pain, the Burton trial [21] did not fit the criteria of testing non-specific low back pain and the Cleary trial [22] did not specify the type of back pain.

One of the difficulties of research in osteopathic medicine where there are only a small number of intervention trials is the lack of consistency in the methodology used. The lack of consensus on what is appropriate methodology remains a substantive barrier to understanding the role of osteopathic medicine in chronic non-specific lower back pain, which forms a major presentation to osteopathic clinical practice. The benefit of consistent methodology is the capacity to better compare clinical trials and where appropriate to use meta-analysis to provide a statistical assessment of a number of smaller homogenous clinical trials grouped together. Failure to develop an effective methodological consensus may leave this question of the effectiveness of osteopathic medicine in non-specific lower back pain unanswered.

A number of the methodological issues have been discussed by researchers in the field [23-26]. These include: the problem of blinding the subject and the treatment provider to the intervention, the subjects’ knowledge and perceptions of the intervention, and the difficulty of control in trials of manual therapy, particularly the credibility of sham treatment. Although both included studies were considered to have a low risk of bias according to the Cochrane Back Review Group [13], both had methodological weakness in blinding of participants and patient compliance, which appear to be common issues in trials of manual therapy interventions [25]. Licciardone and Russo [25] point out that the influence of a number of non-specific treatment effects on clinical outcomes present a major challenge to raising the evidence base of OMT and constructing appropriate clinical trials.

Another methodological issue that arises from this Systematic Review is whether the Randomised Clinical Trial that aims to test efficacy suits a complex intervention like osteopathic medicine in a multifactorial condition like chronic non-specific LBP [24]. The emergence of comparative effectiveness research [26], including the pragmatic trial approach, may point the way to solving the difficulties that researchers have had in meeting the requirements of Evidence Based Medicine and the hierarchy that places Systematic Reviews of rigorous RCTs at its pinnacle.

### Limitations

As in any systematic review, it is possible that there are clinical trials that were not found in the search process. The aim of focussing strictly on CNSLBP limited the number of studies, as many had mixed back pain populations. The requirement of having an authentic osteopathic intervention, which was based on studies of practice in Australia and the United Kingdom, may limit the generalizability of these findings to other jurisdictions.

## Conclusions

In summary, there is a paucity of quality clinical trials testing osteopathic intervention in adult patients with chronic non-specific low back pain, and more data is required. Two trials were included that differed in their conclusions. One trial concluded that the osteopathic intervention was similar in effect to a sham intervention, and the other suggests similarity of the effect of osteopathic intervention to exercise and physiotherapy. Further clinical trials into this subject are required that have consistent and rigorous methods. These trials need to include an appropriate control and utilise an intervention that reflects actual practice.

## Competing interests

All authors declare they have no competing interests.

## Authors’ contributions

PJO and SPM planned the systematic review and developed the methodology. PJO was the principal investigator, completed the literature search, wrote the body of the paper, and was one of the reviewers. SPM was the other reviewer, contributed to the interpretation of the results, edited the paper and co-wrote the discussion. Both authors read and approve the final manuscript.

## Pre-publication history

The pre-publication history for this paper can be accessed here:

http://www.biomedcentral.com/1471-2474/14/129/prepub
